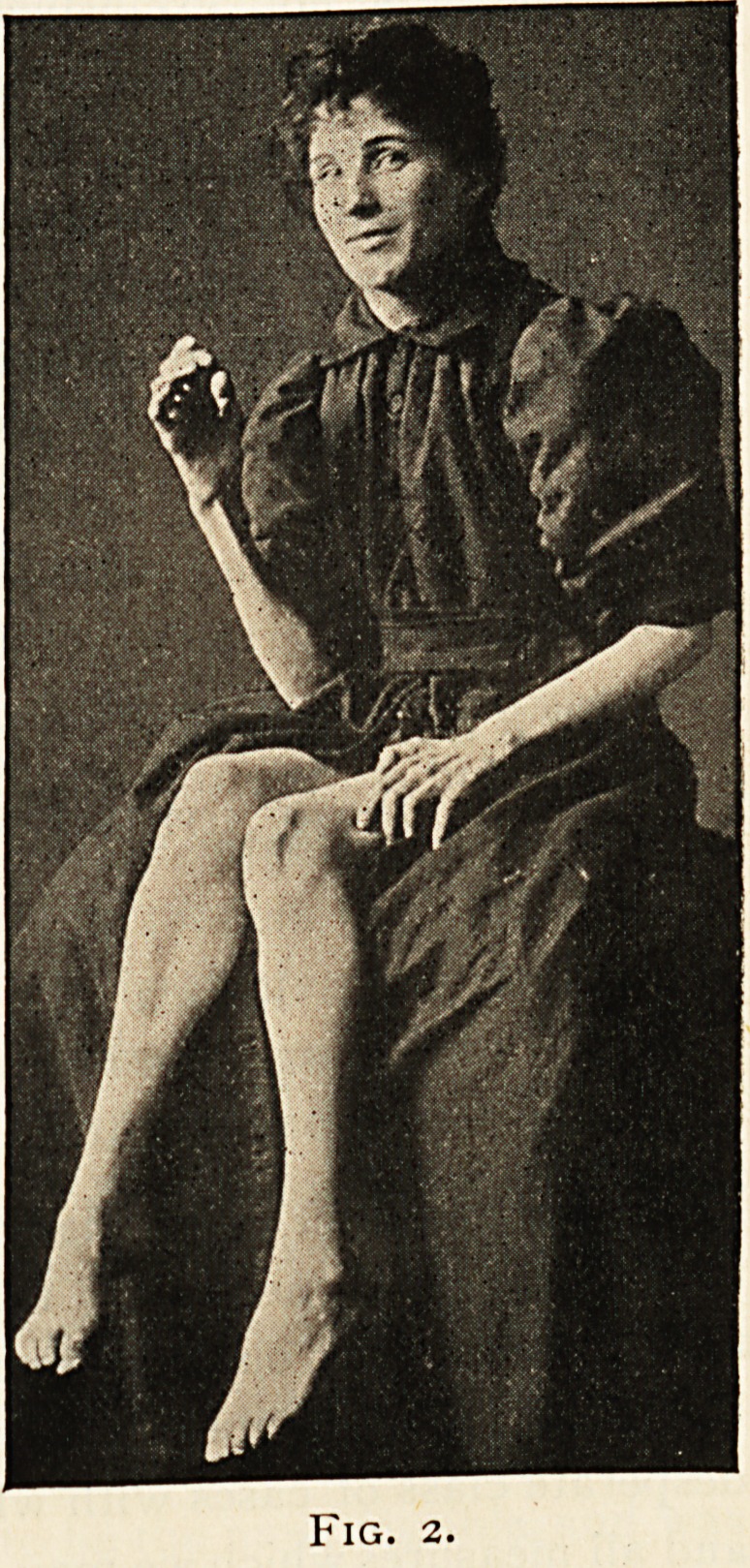# A Case of Neuritic Muscular Atrophy ("Peroneal" Type)

**Published:** 1899-12

**Authors:** J. E. Shaw

**Affiliations:** Professor of Medicine at University College, Bristol; Physician to the Bristol Royal Infirmary.


### A CASE OF NEURITIC MUSCULAR ATROPHY ("PERONEAL" TYPE).

BY

J. E. Shaw, M.B. Ed.,

*Professor of Medicine at University College, Bristol;*
*Physician to the Bristol Royal Infirmary.*

---

To render fully intelligible the complete significance of the illustrations, a brief abstract of the notes upon this case must be given:—

E. G., aged 22, single, formerly a domestic servant, was admitted to the Bristol Royal Infirmary on March 2nd, 1899.

*Family History.*—Father is of a nervous disposition; one sister is also nervous, and another suffered for a long time in childhood from a malady which caused "trembling" and difficulty in walking (chorea?), but no known case of a similar character has occurred in the family.

*Previous History.*—At 5 years of age had a long attack of rheumatic fever, and has had frequent attacks of less severity since. About Christmas, 1893, she observed that her legs were beginning to get weak; three months later she suffered from pain which she distinguished from "rheumatism." and which passed from the right natis down the outer and posterior part of the thigh to the calf. At Whitsuntide, 1894, having further failed in her powers of walking and of getting upstairs, and having noticed that her right foot dragged upon the ground occasionally, she finally gave up her occupation as a domestic servant. She attended the Bath United Hospital, first as an out-patient and afterwards as an in-patient, until November of the same year. While in that institution she observed that her hands were beginning to waste. In the following year she was a patient at the Bath Mineral Water Hospital for nine weeks, without receiving appreciable benefit. During the last four years the complaint has continued to make slow progress.

PRESENT CONDITION.

*Nervous System: Motor.*—There is a great loss of motor power in the legs and thighs; patient can just walk alone, heeling over from side to side in the attempt to lift each foot successively off the ground; notwithstanding, the toes of the right foot always, and those of the left foot occasionally, drag upon the floor; the gait is not high-stepping, on account of the weakness of the thigh muscles. There is no impairment of the action of the sphincters. The hands and fore-arms are extremely feeble, and the fingers cannot be completely extended or closed—the patient manages, however, to use a spoon and fork. The upper arms are slightly affected: the shoulders, neck, and back not at all. There is defective action of the right side of the face, and the tongue on being protruded deviated strongly to the right. (See Fig. 1.)

*Sensory.*—There is decided (but not total) anæsthesia of all forms in both feet, most marked in the right foot; a less degree of anæsthesia

FIG. 1.

of the legs, particularly of the anterior surfaces; slight anæsthesia, superficial to the vasti interni. The hands also are distinctly anæsthetic to all forms of sensation, but less so than the feet. The fore-arms are slightly anæsthetic, the upper arms apparently not so at all. There is no anæsthesia of the face.

*Reflexes.*—Knee-jerks, plantar reflexes, ankle- and rectus-clonus are all completely absent.

*Trophic.*—The extensor brevis digitorum of each foot is quite atrophied, and apparently also the interossei and plantar muscles, although of this it is impossible to be certain. There is considerable atrophy of the muscles on the anterior surface of the legs, leading to marked foot-drop (see Fig. 2); the muscles on the posterior aspect of the legs are also atrophied, although not excessively so; the muscles on the anterior and inner surfaces of the thighs are also wasted. In the hands the whole of the intrinsic muscles appear to be completely atrophied; the muscles in the fore-arms, including the supinator longus, are also much diminished in bulk, the extensors particularly so: the upper-arm muscles are but slightly affected, and the shoulder muscles not at all so. There is no fibrillary tremor.

*Electrical Reaction.*—There is no response to the interrupted current in either foot, leg, or thigh, except in the vastus externus, which reacts slightly to a strong current. Electro-sensibility is much impaired, though not destroyed, in these same regions. Similarly there is no response in the hand and fore-arm muscles; the biceps acts slightly, the deltoid and pectoralis major normally. Electro-sensibility in the hands and fore-arms is slightly diminished.

FIG. 2.

The other systems presented no phenomena of importance. Six weeks after admission patient suffered an exacerbation of her condition. She complained of pains down her legs and arms, which upon investigation were found to be seated in the nerve-trunks. These continued for three or four days, and were followed by increased feebleness of the extremities, increased lateral deviation of the tongue, and some difficulty of articulation and deglutition. From this condition she made some improvement, but three weeks later had another attack of the same nature: this left her so feeble that she could not stand alone, or turn herself in bed, or feed herself; her speech was almost unintelligible from defective lingual and palatal action, mucus accumulated in her pharynx, and occasional regurgitation through the nares occurred upon swallowing liquids. From this second exacerbation she had recovered to a considerable degree when she returned to her home a week or two later.

The case is interesting and perhaps somewhat unusual, inasmuch as while on the one hand it resembled in many respects a case of ordinary progressive muscular atrophy, with bulbar symptoms becoming developed as the case progressed, on the other hand the sensory phenomena, the condition of the reflexes and of the electrical reaction show indisputably that the lesion was not exclusively myopathic or even situated in the nerve-centres, but was partly, if not wholly, seated in the peripheral nerves. Possibly the case was an example of the combined form of which Sir William Gowers speaks, but the complete absence of Faradic response in the considerable volume of calf-muscles which still existed (see Fig. 2) is in itself a proof that the process was not exclusively seated in the muscles.

## Figures and Tables

**Fig. 1. f1:**
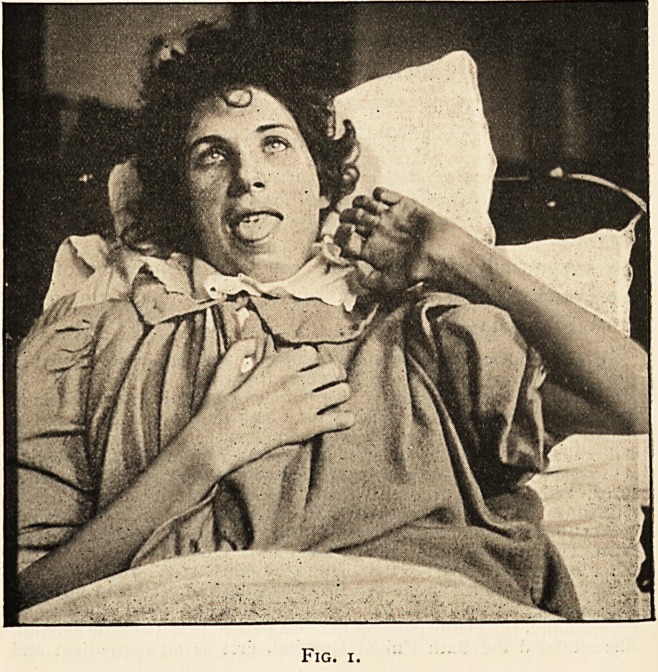


**Fig. 2. f2:**